# Implementing structured follow-up of neonatal and paediatric patients: an evaluation of three university hospital case studies using the functional resonance analysis method

**DOI:** 10.1186/s12913-022-07537-x

**Published:** 2022-02-14

**Authors:** Véronique Bos, Daniëlle Roorda, Eleonore de Sonnaville, Menne van Boven, Jaap Oosterlaan, Johannes van Goudoever, Niek Klazinga, Dionne Kringos

**Affiliations:** 1 Department of Public and Occupational Health, Amsterdam UMC, University of Amsterdam, Amsterdam Public Health Research Institute, Amsterdam, Netherlands; 2 Department of Pediatric Surgery, Emma Children’s Hospital, Amsterdam UMC, University of Amsterdam and Vrije Universiteit, Amsterdam Reproduction and Development, Amsterdam, Netherlands; 3 Pediatric Intensive Care Unit, Emma Children’s Hospital, Amsterdam UMC, University of Amsterdam, Amsterdam, Netherlands; 4 Neonatal Intensive Care Unit, Emma Children’s Hospital, Amsterdam UMC, University of Amsterdam, Amsterdam, Netherlands; 5 Follow Me Programme and Emma Neuroscience Group, Department of Pediatrics, Emma Children’s Hospital, Amsterdam UMC, University of Amsterdam, Amsterdam Reproduction and Development, Amsterdam, Netherlands

**Keywords:** Follow-up, Implementation science, Quality improvement, Long-term outcomes, Functional resonance analysis method (FRAM), Neonatal intensive care, Paediatric intensive care, Paediatric surgery

## Abstract

**Background:**

In complex critical neonatal and paediatric clinical practice, little is known about long-term patient outcomes and what follow-up care is most valuable for patients. Emma Children’s Hospital, Amsterdam UMC (Netherlands), implemented a follow-up programme called Follow Me for neonatal and paediatric patient groups, to gain more insight into long-term outcomes and to use such outcomes to implement a learning cycle for clinical practice, improve follow-up care and facilitate research. Three departments initiated re-engineering and change processes. Each introduced multidisciplinary approaches to long-term follow-up, including regular standardised check-ups for defined age groups, based on medical indicators, developmental progress, and psychosocial outcomes in patients and their families. This research evaluates the implementation of the three follow-up programmes, comparing predefined procedures (*work-as-imagined*) with how the programmes were implemented in practice (*work-as-done*).

**Methods:**

This study was conducted in 2019–2020 in the outpatient settings of the neonatal intensive care, paediatric intensive care and paediatric surgery departments of Emma Children’s Hospital. It focused on the organisational structure of the follow-up care. The functional resonance analysis method (FRAM) was applied, using documentary analysis, semi-structured interviews, observations and feedback sessions.

**Results:**

One work-as-imagined model and four work-as-done models were described. The results showed vast data collection on medical, developmental and psychosocial indicators in all work-as-done models; however, process indicators for programme effectiveness and performance were missing. In practice there was a diverse allocation of roles and responsibilities and their interrelations to create a multidisciplinary team; there was no one-size-fits-all across the different departments. Although control and feedback loops for long-term outcomes were specified with respect to the follow-up groups within the programmes, they were found to overlap and misalign with other internal and external long-term outcome monitoring practices.

**Conclusion:**

Implementing structured long-term follow-up may provide insights for improving daily practice and follow-up care, with the precondition of standardised measurements. Lessons learned from practice are (1) to address fragmentation in data collection and storage, (2) to incorporate the diverse ways to create a multidisciplinary team in practice, and (3) to include timely actionable indicators on programme effectiveness and performance, alongside medical, developmental and psychosocial indicators.

**Supplementary Information:**

The online version contains supplementary material available at 10.1186/s12913-022-07537-x.

## Background

To date, data collection in healthcare systems has been predominantly designed to obtain short- and intermediate-term data on treatment outcomes for patients. The standardised collection of long-term outcome data and data on follow-up care is faced with many challenges; it is therefore often absent [1, 2]. As pressures increase to maintain and improve the health of populations, the objective of high-value healthcare systems, as described by Porter, has gained importance on national policy agendas [3–5]. Supported by Donabedian’s model of structure, process and outcome, a standardisation of outcome measurements is needed to gain necessary insights for improvement [6, 7].

The field of critical neonatal and paediatric clinical practice has experienced substantial improvements in short-term survival and mortality rates [8–11]. In light of the higher short-term survival rates, a shift of focus towards long-term outcomes is now in progress, in order to support children with adequate follow-up care. Although regular medical follow-up of patients is already taking place in many patient groups, systematic data collection and standardised protocols for follow-up care are often missing. Follow-up programmes can be used to standardise content in follow-up practices and to systematically collect data on long-term outcomes, in order to provide the insights needed to aid decision-making on treatment and follow-up care [12, 13]. In rare and complex paediatric patient groups, disease and treatment may have effects on children’s development, and advances in treatment are moving at a rapid pace [14]. Hence, systematic data collection in rare paediatric patient groups needs to be more comprehensive and to encompass long-term medical, developmental and psychosocial indicators [15–17]. For this reason, a follow-up programme was implemented at Emma Children’s Hospital, Amsterdam UMC (Netherlands), targeting three departments with high-cost, highly complex patient groups: (1) the neonatal intensive care units (NICUs), (2) the paediatric intensive care unit (PICU), and (3) the paediatric surgery department (patients who had undergone neonatal surgical treatment for congenital malformations).

Re-engineering and change processes in daily clinical practices are known to be challenging. By evaluating the three implemented follow-up programmes, this research aims to learn from daily practice and to improve the quality and sustainability of the follow-up programmes as well as their impact on clinical practice. Our research objective is to evaluate the implementation of the outpatient follow-up programmes of the neonatal ICUs, paediatric ICU and paediatric surgery departments by comparing work in practice (*work-as-done*), with the mission, vision, organisational blueprint and tools established by the management team of the Follow Me programme (*work-as-imagined*). The following questions are addressed: (1) How is the implemented follow-up programme (a) generating data collection and standardisation for the measurement of long-term outcomes, (b) allocating key stakeholder roles and responsibilities, and (c) building control and feedback loops to aid decision-making? (2) Can gaps between work-as-imagined and work-as-done be identified in the three case studies and, if so, how could such gaps be overcome using within- and cross-case-study learning?

## Methods

The study conforms to the Consolidated Criteria for Reporting Qualitative Studies (COREQ, Additional file 1).

### Setting

The work-as-imagined model is based on the Follow Me programme of Emma Children’s Hospital, Amsterdam UMC. The implementation of the Follow Me programme was the responsibility of one overarching project team and adapted to the different needs of the patient groups and departments by the responsible manager of the department and a coordinator specifically for the Follow Me programme per department. The four work-as-done models are based on the observed outpatient settings connected to the two neonatal ICUs, the paediatric ICU and the paediatric surgery department of Emma Children’s Hospital. The neonatal ICU follow-up already existed for preterms (< 30 weeks GA), dysmatures (< 1500 g) and children with neurological abnormalities, but the Follow Me programme added standardized structured data collection and evaluation sessions. The paediatric ICU already offered regular follow-up with the paediatric intensivist and the psychologist, but the Follow Me programme added standardized structured data collection and evaluation sessions. The paediatric surgery follow-up of patients with surgical congenital malformations (e.g. esophageal atresia, abdominal wall defects, pulmonary airway malformations, sacrococygeal teratoma, anorectal malformations, Hirschsprung disease and short bowel syndrome) focussed mainly on functional gastrointestinal or pulmonary outcomes, but surgical treatment at infant age may also impact growth, somatic, psychosocial and neurodevelopmental outcomes. The Follow Me programme added standardized holistic data collection and evaluation sessions including these outcomes. In Table 1 an overview of the three different departments is given regarding follow up time points (or ages), size of clinical setting, and number of patients attending the follow-up programme. As a result of a recent merger between two university medical centres, neonatal intensive care still had two separate post-ICU outpatient clinics, at the locations Academic Medical Centre (AMC) and VU University Medical Centre (VUMC); work-as-done for neonatal intensive care was therefore studied at both locations.

**Table 1:** Case study descriptions

Case study/ department	Follow Me programme launched	Outpatient follow-up time points or ages^a^	Number of clinical patients treated in 2019	Number of unique patients with an appointment in the follow-up programme in 2019
Neonatal ICUs	2020	6, 12 and 24 months and 5 and 8 years of age	Location AMC: ~ 400 unique neonates Location VUMC: ~ 360 unique neonates	Location AMC: ~ 500^b^ Location VUMC: ~ 250^b^
Paediatric ICU	March 2018	3-to-6 and 12 months after ICU discharge; 6 years of age	564	92
Paediatric surgery department	October 2017	6, 12 and 24 months and 5, 6, 8, 12 and 16^c^ years of age	46 infants born with congenital disorders in 2019	94

^a^follow-up time points were chosen in accordance to research (e.g. PICU time point 6 years of age due to validity of lung tests), national standards (e.g. NICU time points), important steps in motor development (e.g. time point 12 months learning to walk) and patient or their families need (e.g. time point 6 years of age transition to school). ^b^Children in follow-up before the Follow Me programme was launched ^c^ Before 2019, age 17

### Research design

The functional resonance analysis method (FRAM) was applied (see next section). Documentary analysis, semi-structured interviews, observations and feedback sessions were conducted to identify key *functions* and their *aspects* and *interrelations*, enabling us to model and compare work-as-imagined (predefined procedures of the Follow Me programme) with work-as-done (work in practice in the three outpatient departments of the neonatal ICU, paediatric ICU and paediatric surgery). The scope of the models was the organisational context of the follow-up programme in terms of (a) generating data collection and standardisation for the measurement of long-term outcomes, (b) allocating key stakeholder roles and responsibilities, and (c) building control and feedback loops to aid decision-making. Close collaboration between researchers and the staff participating in the follow-up programmes was established in order to tailor the research to the contexts of the case studies, to accurately model the results, and to stimulate mutual learning and improvement by capitalising on experiences from practice and on research findings.

### The functional resonance analysis method (FRAM)

**Fig. 1:**
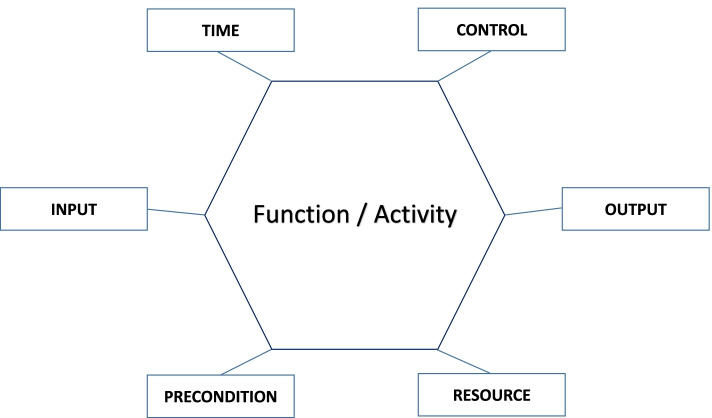
FRAM hexagon function with aspects

The FRAM method is described by Hollnagel and colleagues [18, 19]. It is a systematic, participatory approach designed to model how complex processes usually go right in practice (*work-as-done*) and to compare work-as-done with predefined procedures (*work-as-imagined*) [20–23]. FRAM models the work-as-done by pinpointing relevant *functions*. Functions are defined as ‘the activities – or set of activities – that are required to produce a certain outcome’ [18]. Functions are displayed in hexagons (Fig. 1) and then described using six aspects: *Input* (I), *Output* (O), *Preconditions* (P), *Resources* (R), *Control* (C) and *Time* (T) (Table 2). Potential *couplings* between functions are added by identifying shared attributes of aspects. Such couplings represent possible relationships or dependencies between functions, but do not refer to any particular situation. Not all aspects of each function need couplings. Couplings are generally *n*-to-*n* rather than 1-to-1 [22].

**Table 2:** Aspects of FRAM functions

Aspect	Description	Examples from the Evaluate Outcomes function in the work-as-imagined model
Input	What activates the function	Initiated by project team
Output	The result of the function	New insights into long-term outcomes of disease or treatment; verification of completeness of data or coverage of follow-up programme; critical reflection on outcomes by multidisciplinary team
Precondition	What needs to be verified before the function is carried out, but does not activate the function itself	No coupling observed
Resource or execution condition	What is consumed by the function while the function is active. A Resource diminishes while function is active, but an execution condition does not	Patient data from follow-up, patient-reported outcome measurement (PROM), multidisciplinary team, patient participation, patient satisfaction data, patient data from initial treatment
Control	What supervises or regulates the function in order to produce the desired Output	No coupling observed
Time	Temporal relations between one function and others; in some cases, Time can also be seen as a Precondition, Resource or Control aspect	Yearly evaluation and improvement sessions

### Data collection and coding

Our data collection took place from September 2019 to July 2020. An overview of collected data and data use per model can be found in Additional file 3. To gather data on the work-as-imagined, we collected internal protocols and management documents on the Follow Me programme(*n* = 16) and composed and conducted semi-structured interviews (*n* = 10) with all members of the overarching project team of the Follow Me programme (*n* = 4), with the responsible departmental manager per department (*n* = 3), and with the responsible coordinator for the Follow Me programme per department (*n* = 3). The interviewees gave informed consent to the research, and interviews were audio-recorded. To obtain relevant data from the interviews, we used as an interview guide the set of standard questions from the FRAM methodology as proposed by Hollnagel and colleagues [19].

To gather data on the work-as-done, we conducted at least 2 observations of full outpatient follow-up visits for every case study (total *n* = 8), studied work documents on outcomes of the follow-up programmes (*n* = 2), and conducted observations during an evaluation session on outcomes per case study, if held during the study period (*n* = 2). Informed consent to the research was obtained from the patients and their families, and from health care providers involved in the observations, before the start of the outpatient follow-up visits. A template for note-taking was used during observations, focused on identifying functions, aspects and interdependencies (see template in Additional file 2).

The first author coded the collected data into functions, in line with the Hollnagel handbook (19). Related aspects (Input, Output, Preconditions, Resources, Control and Time) were added to the functions, and potential couplings were drawn.

### Modelling and calibration of models

FRAM models visualize what happens in imagination or practice by studying activities or functions, their aspects and interrelations. The list of identified functions, aspects and potential couplings per model was then mapped to identify those functions and aspects that affected the three organisational scope themes ((a) generating data collection and standardisation for the measurement of long-term outcomes, (b) allocating key stakeholder roles and responsibilities, and (c) building control and feedback loops to aid decision-making). If one such theme applied to a function or aspect, that function or aspect was defined as a *foreground* function or aspect and shown in the models. Background functions judged as not having that potential were not shown in the models. The FRAM Model Visualizer (FMV) software, version 2.1.0, was used to shape the models (24).

FRAM improvement sessions per department (*n* = 3) were organised to validate findings. Only one content change was made to the models following those sessions: a separation of patient-reported outcome measurement (PROM) routes for (1) psychosocial indicators and (2) medical indicators in the paediatric surgery model. Some visual changes were also made, such as placement of key stakeholders separately in the model to highlight them.

### Analysis of the models: learning within and across case studies

A descriptive analysis of the models was made which defined human, organizational, and environmental variability, and upstream and downstream interdependence of the foreground functions. The identified gaps between the work-as-imagined and work-as-done models in relation to the three organisational scope themes (a, b and c) both within and across case studies were drafted by VB and discussed with the Follow Me project team. Improvement sessions with each department were organised to discuss the findings, define if the gaps were facilitating or hindering factors, and highlight areas for improvement actions per department and/or for the Follow Me programme as a whole.

## Results

A total of five models were constructed: one work-as-imagined model for the Amsterdam UMC Follow Me programme and four work-as-done models, two for the neonatal ICUs (Locations AMC and VUMC), one for the paediatric ICU and one for the paediatric surgery department.

### Work-as-imagined model in the Follow Me programme

**Fig. 2:**
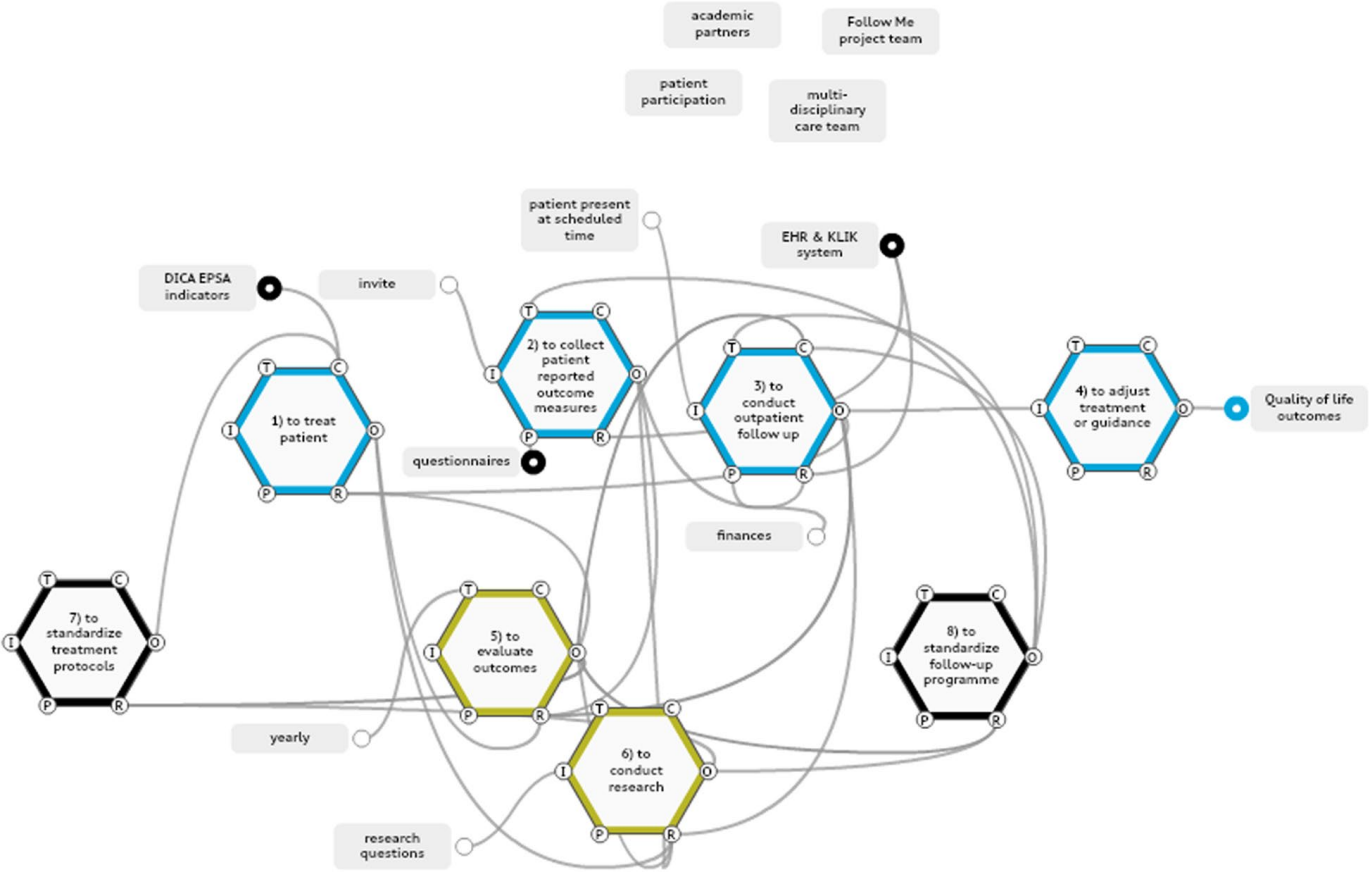
Work-as-imagined model Follow Me programme. Blue: data collection, black: standardization of treatment and follow-up, yellow: controls & feedback loops. Key stakeholders are extracted from the model and shown seperately in the top right corner

In the analysis of work-as-imagined in the Follow Me programme, 18 functions were identified, of which 8 were selected as foreground functions relating to (a) generating data collection and standardisation for the measurement of long-term outcomes, (b) allocating key stakeholder roles and responsibilities, and (c) building control and feedback loops to aid decision-making (Fig. 2).

### Data collection and standardisation

Standardisations of data measurement instruments were considered to be Preconditions for evaluating outcomes (function 5 in Fig. 2) and for conducting research (function 6 in Fig. 2). In turn they were to enable creation of a knowledge base on disease, treatment and follow-up outcomes. The functions that generated data collection and their aspects are shown in Additional file 4.

### Allocation of key stakeholder roles and responsibilities

The key stakeholder in the work-as-imagined model was the patient, surrounded by a multidisciplinary team providing standardised follow-up care and personalised care. Patient organisations were to have a Control, or monitoring, aspect in maintaining a patient-oriented approach. For example patient organizations were asked to reflect on the proposed protocol for the follow-up programmes and invited to attend the yearly evaluation sessions in which the results of the Follow Me programme were presented and areas for improvement were discussed. Academic centres had a Control aspect in benchmarking treatment outcomes and were seen as learning partners in these complex, often rare, patient groups. A dedicated project team for the Follow Me programme initiated outcome evaluation sessions.

### Building control and feedback loops

Mechanisms for the multidisciplinary team to receive and respond findings of the programme were established via multiple control and feedback loops. The functions (5) to evaluate outcomes, (6) to conduct research, (7) to standardise treatment protocols and (8) to standardise the follow-up programme were to be used to build control and feedback loops designed to improve treatment and follow-up protocols. Preconditions within feedback loops were the systematic collection of standardised PROMs and the performance of standardised measurements and data collection on medical, developmental and psychosocial outcomes by the multidisciplinary team during the follow-up. To establish and continuously improve follow-up protocols, the standardisation process of the follow-up programme was to make use of research output, evaluations of follow-up outcomes, the experience of the multidisciplinary team, and the cooperative relationships with patient organisations and with other academic centres. Such interrelations formed a cycle of collection of long-term outcomes, and these were to feed into the insights that would provide feedback on treatment and follow-up protocols.

### Work-as-done at the neonatal ICU, Location AMC

**Fig. 3:**
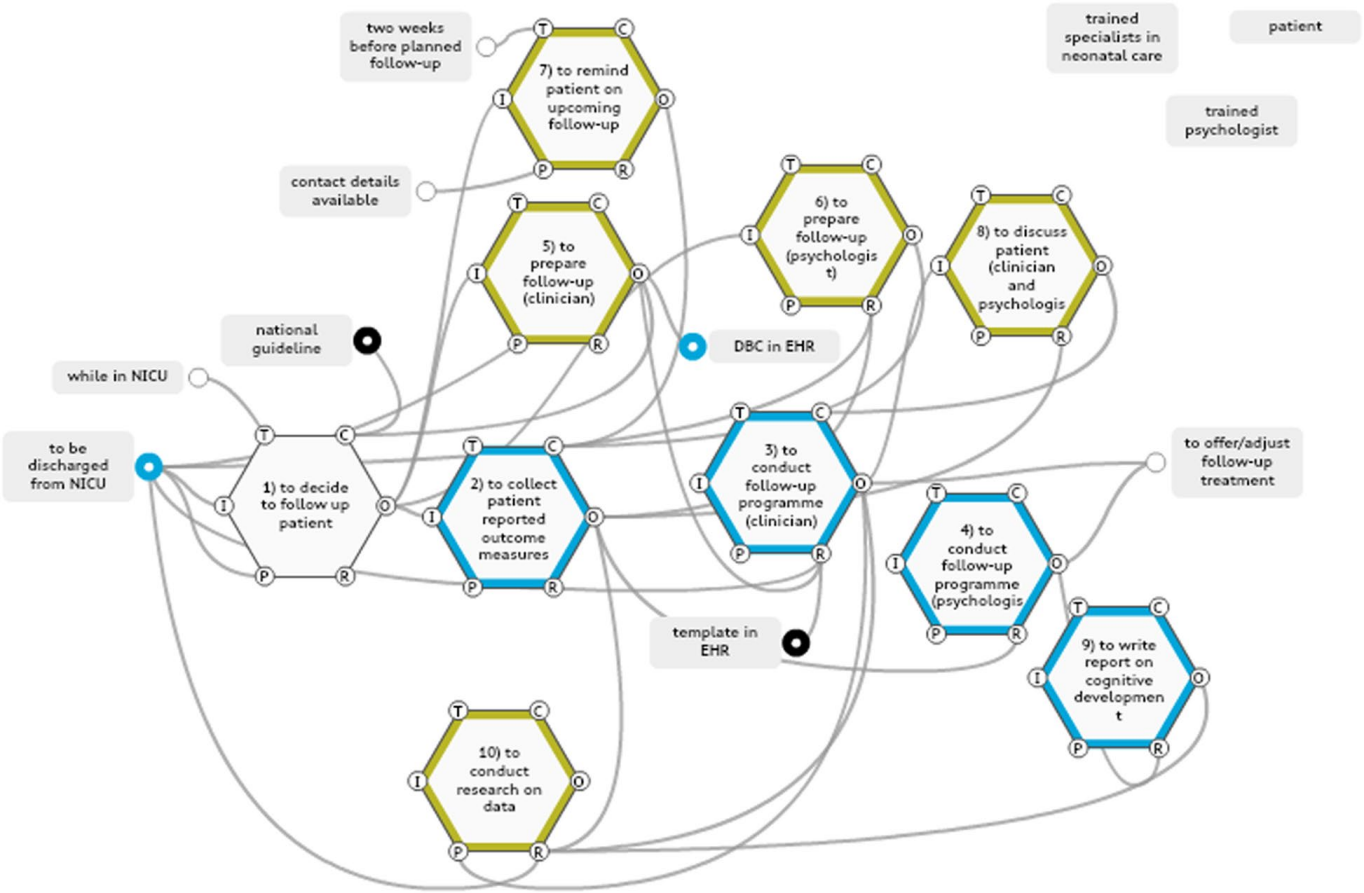
Work-as-done model neonatal ICU, location AMC. Blue: data collection, black: standardization of treatment and follow-up, yellow: controls & feedback loops. Key stakeholders are extracted from the model and shown seperately in the top right corner

In the analysis for the work-as-done model of the AMC neonatal ICU, 12 functions were identified, of which 10 were selected to be foreground functions relating to (a) generating data collection and standardisation for the measurement of long-term outcomes, (b) allocating key stakeholder roles and responsibilities, and (c) building control and feedback loops to aid decision-making (Fig. 3).

### Data collection and standardisation

Standardisation of data measurement instruments, including standardised data collection time points, was based on national guidelines in place since the 1990s [12]. The functions that generated data collection and their aspects are shown in Additional file 4.

### Allocation of key stakeholder roles and responsibilities

A patient defined by the national guidelines as belonging to an at-risk group was offered the standardised follow-up programme in two parts, one with the neonatologist and one with the psychologist. There was an alignment between the neonatologist and the psychologist, with their functions interlinked by consecutive time slots and patient handover. A patient could receive additional or adjusted care, referral or guidance from both the neonatologist and the psychologist individually.

### Building control and feedback loops

In this model, small feedback loops were seen that monitored previous functions or Preconditions for downstream functions. In addition, the face-to-face handover between the neonatologist and the psychologist incorporated a Control aspect to make sure a consent form for data use for research purposes had been signed and collected (a Precondition for conducting research). Standardised regular internal evaluation sessions on the outcomes of follow-ups did not take place at the AMC neonatal ICU at the time of this research, and were therefore not modelled.

### Work-as-done at the neonatal ICU, Location VUMC

**Fig. 4:**
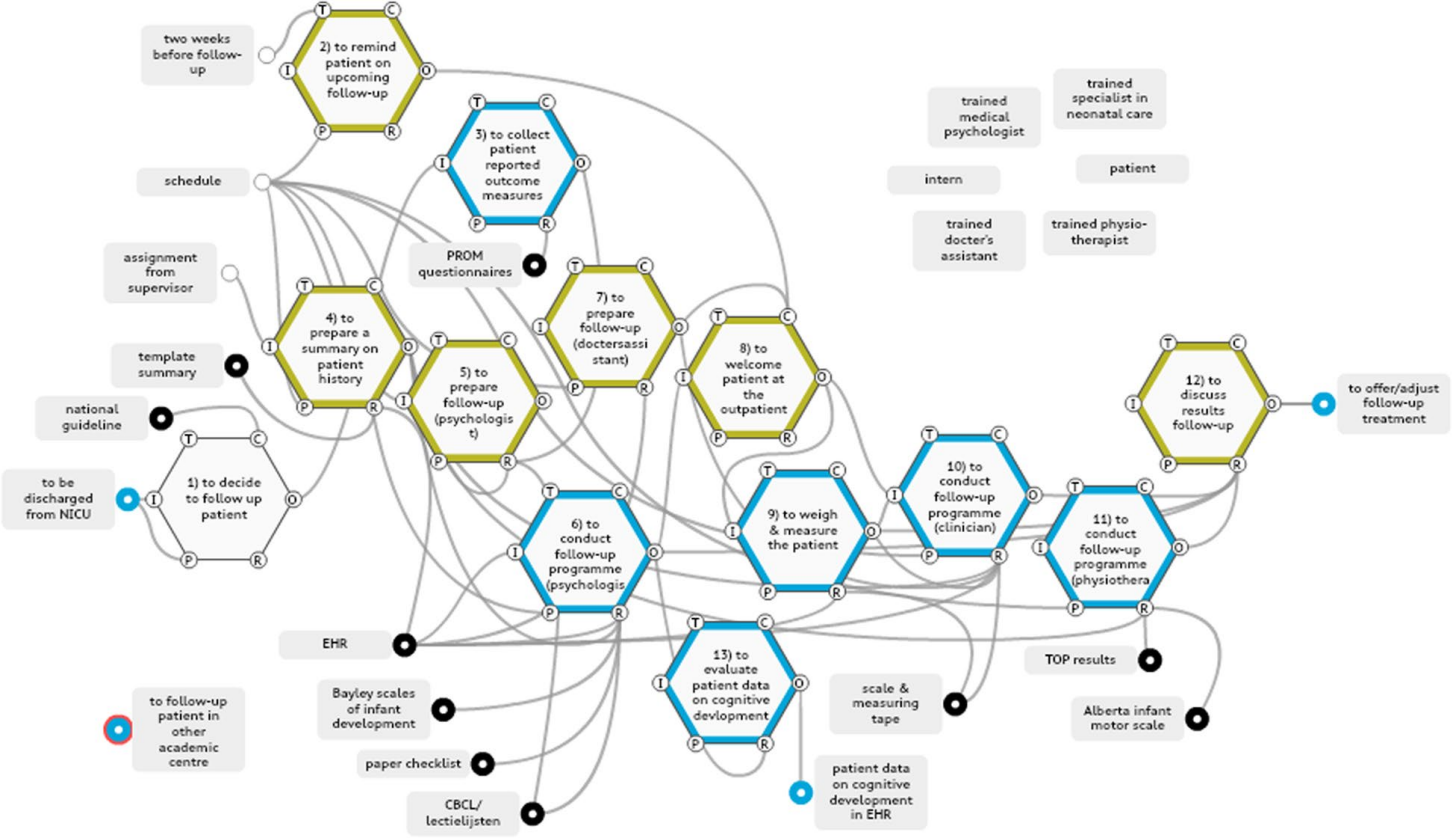
Work-as-done model neonatal ICU, location VUMC. Blue: data collection, black: standardization of treatment and follow-up, yellow: controls & feedback loops. Key stakeholders are extracted from the model and shown seperately in the top right corner

The analysis for the work-as-done model of the neonatal ICU, Location VUMC, included 17 functions, of which 13 were defined as foreground functions relating to (a) generating data collection and standardisation for the measurement of long-term outcomes, (b) allocating key stakeholder roles and responsibilities, and (c) building control and feedback loops to aid decision-making (Fig. 4).

### Data collection and standardisation

Standardisation of data measurement instruments, including standardised data collection time points, was based on national guidelines in place since the 1990s [12]. All the health care providers involved prepared the follow-up separately, but were aided by a summary of the patient’s history constructed by a medical psychology trainee with the help of a template. That summary was only available to all the involved health care providers if cognitive development was also assessed (not done in all age groups). The functions that generated data collection and their aspects are shown in Additional file 4.

### Allocation of key stakeholder roles and responsibilities

Patients defined by the national guidelines as belonging to an at-risk group were offered the standardised follow-up care involving four consultations. The first was with the psychologist (if the patient was in the age group offered assessment of cognitive development) in a separate but nearby outpatient area. Subsequent sessions were planned with the medical assistant, the neonatologist and the physiotherapist in another outpatient area. All consultations were scheduled consecutively, thus minimising patient waiting time. The follow-up care was coordinated by a designated medical assistant with a host function towards the patient. The assistant welcomed the patient and provided guidance on which questions to pose to which health care provider. In case the next planned provider was not ready to conduct the follow-up when the patient was available, the assistant tried to switch around consultations, thus reducing delays for the patient and providers alike. The assistant also provided brief patient handovers in person to the health care providers, highlighting certain concerns the patient might have and giving an update on consultations so far. The role and responsibilities of the host function were confined to the follow-up programme within the department. In our observations, patients who had received treatment in two different academic neonatal ICUs during hospital stays would now be attending two similar follow-up programmes, one in each academic centre, as both centres were compliant with national guidelines. There was a stronger interlinkage between the neonatologist and the physiotherapy disciplines; those functions were partially performed simultaneously in the same room. This facilitated communication between the two professions and allowed for the parents of the patient to be questioned by the intensivist, while the physiotherapist observed the infant or child in terms of motor development.

### Building control and feedback loops

A Control function was created that reminded the patient of the follow-up and the PROMs questionnaire to be completed two weeks beforehand. The prepared summary and the preparation functions for each follow-up function involving health care professionals (functions 6, 9, 10 and 11) served as Controls for the data availability needed for the follow-up programme. Individual results from the professionals’ follow-ups were discussed in a multidisciplinary meeting straight after the follow-ups to align thoughts on future treatment. Standardised regular internal evaluation sessions on the outcomes of follow-ups were not being conducted in the VUMC neonatal ICU department at the time of this research, and were therefore not modelled.

### Work-as-done in the paediatric ICU follow-up

**Fig. 5:**
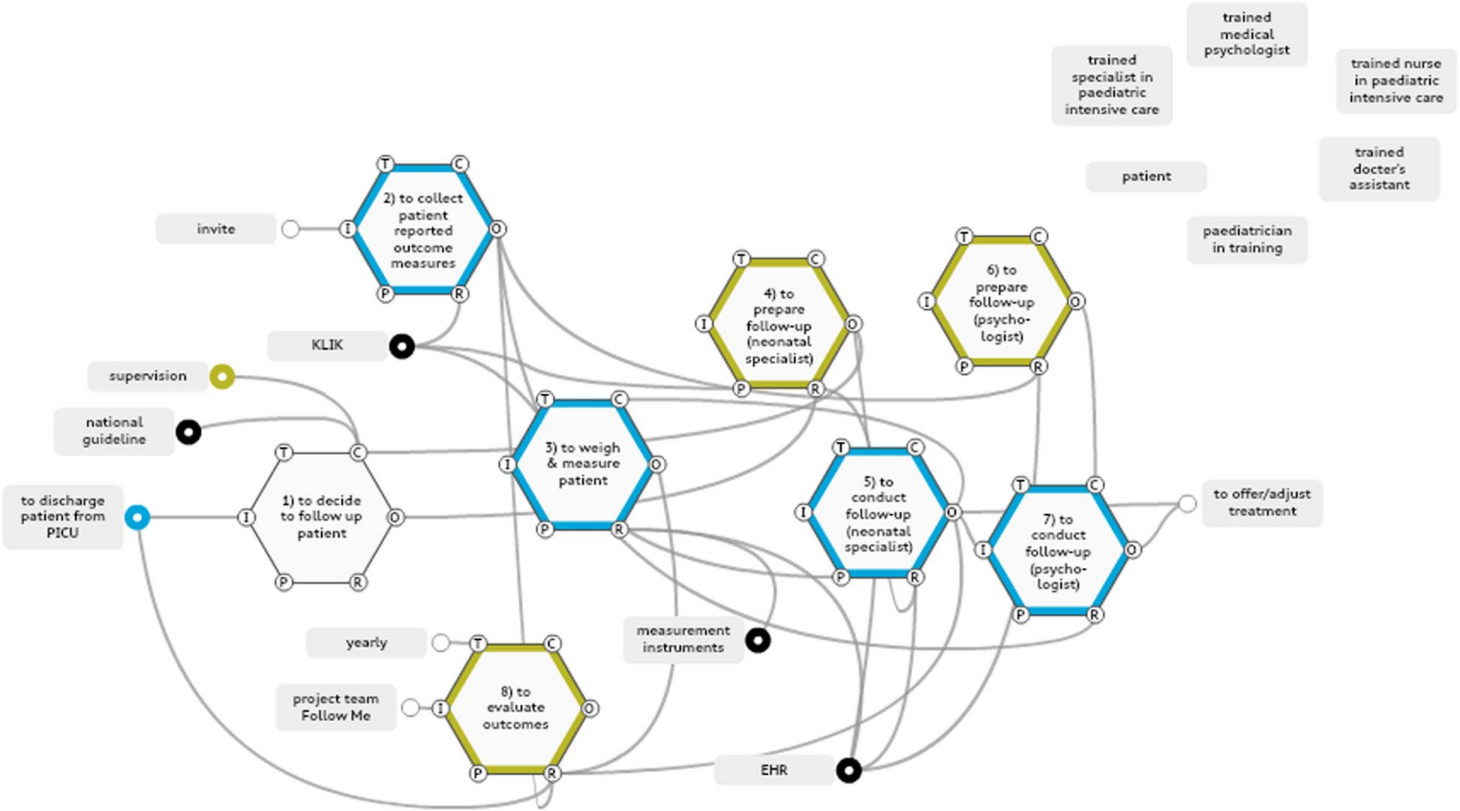
Work-as-done model paediatric ICU. Blue: data collection, black: standardization of treatment and follow-up, yellow: controls & feedback loops. Key stakeholders are extracted from the model and shown seperately in the top right corner

The analysis for the paediatric ICU’s work-as-done model included 11 functions, of which 8 were defined as foreground functions relating to (a) generating data collection and standardisation for the measurement of long-term outcomes, (b) allocating key stakeholder roles and responsibilities, and (c) building control and feedback loops to aid decision-making (Fig. 5).

### Data collection and standardisation

Standardisation of the follow-up programme was based on the national guidelines for follow-up after paediatric intensive care [13]. Patients had problems with delivering the patient-reported outcomes in time for the follow-up programme; in one of our observations, four out of four patient families had failed to meet that goal. That resulted in fewer Resources for the psychologist to prepare for the follow-up and could lead to inadequate Resources or bias for outcome evaluation and research. In our observations, patients reported receiving the invitation to the online portal for the PROM questionnaires on paper, whereas they preferred digital invites to access the questionnaires directly by clicking on a link whilst at the computer. Another observed problem was confusion amongst the parents of a patient about which questionnaire was needed for the current follow-up; they referred to other evaluation questionnaires received after the hospitalisation and a questionnaire from a children’s support service outside the scope of the department. All data on medical indicators were collected using standardised templates in the patient EHR. The functions that generated data collection and their aspects are shown in Additional file 4.

### Allocation of key stakeholder roles and responsibilities

The modelled work-as-done started during the discharge procedure from the paediatric intensive care unit. With the help of a decision tree, a decision was made by a paediatrician-in-training, supervised by a paediatric intensivist. In addition to patients included in line with the decision tree, other patients might be included in the follow-up programme by exception on advice of clinicians and nurses who had treated the patient. If a positive decision was made, the patient was offered the standardised follow-up in three parts, first seeing the medical assistant of the outpatient clinic, then the paediatric intensivist, and then the psychologist. If necessary, a paediatric pulmonologist, cardiologist, neurologist or neuropsychologist could be included in the follow-up, but such consultations were not needed for the patients we observed. Key stakeholders were the paediatric intensive care specialist and the psychologist. The interlinkage between the functions of these stakeholders was reinforced by close proximity, consecutive time slots and face-to-face patient handovers. In our observations, however, a face-to-face handover was not always possible due to misalignments of available time caused by delays in consultations.

### Building control and feedback loops

Both the paediatric intensivist and the psychologist prepared for the follow-up in order to ensure Preconditions for the physical follow-up with the patient. No Time aspect interlinked these functions, however; hence, if necessary information, such as PROM results, was found to be missing, there was no opportunity to rectify that in time for the follow-up consultations. One patient in our observations had undergone a lung function test the week before the follow-up, but the test results from the pulmonologist were scheduled for after the follow-up clinic visit. This suggests possible misalignment between the follow-up programme and routine medical check-ups. The observations detected no Control function to check alignment with routine medical follow-up procedures. The Follow Me programme in the paediatric ICU convened a yearly improvement session, attended by all the participating health care providers, patient organisations and the departmental management team, to evaluate outcomes, protocols and organisational indicators.

### Work-as-done model in the paediatric surgery follow-up

**Fig. 6:**
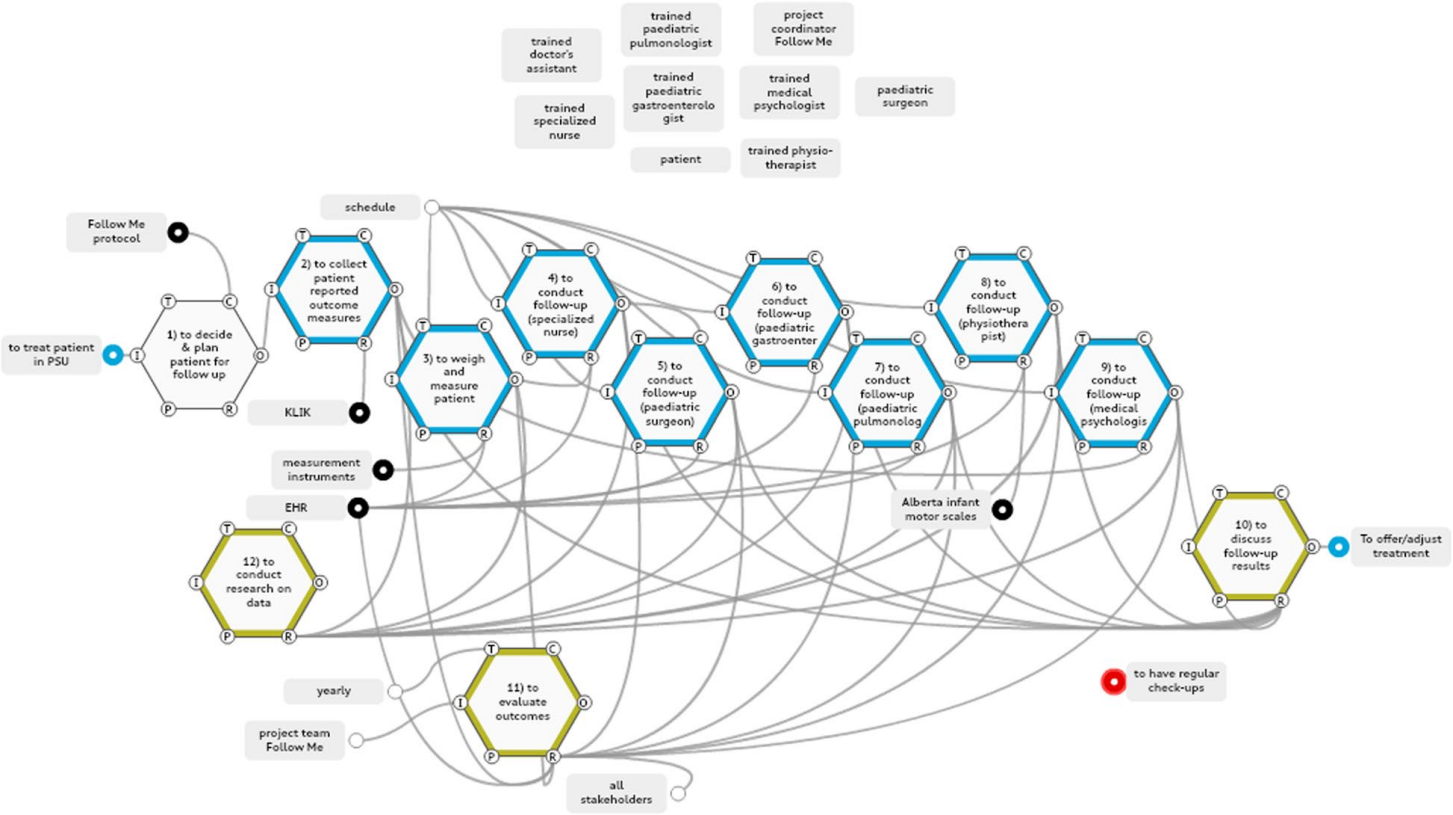
Work-as-done model paediatric surgery department. Blue: data collection, black: standardization of treatment and follow-up, yellow: controls & feedback loops. Key stakeholders are extracted from the model and shown seperately above the model

The analysis for the work-as-done model in the paediatric surgery department’s outpatient clinic identified 15 functions, of which 12 were defined as foreground functions relating to (a) generating data collection and standardisation for the measurement of long-term outcomes, (b) allocating key stakeholder roles and responsibilities, and (c) building control and feedback loops to aid decision-making (Fig. 6).

### Data collection and standardisation

The time spent between patient and health care providers was largely being used for data collection purposes. One family reported during our observations that it was not clear to them whether PROMs about parental psychological outcomes were available to specific health care providers; as the PROMs included personal information on psychosocial factors involving the parents, they found it odd not to know who was aware of that information. PROMs on psychosocial factors were in fact reviewed by the psychologist only, who drew on them to provide input in multidisciplinary meetings after the outpatient visit. Other health care providers did have access to the PROMs before the outpatient care began, but, in our observations, use was not made of that access. The functions that generated data collection and their aspects are shown in Additional file 4.

### Allocating key stakeholder roles and responsibilities

The standardised follow-up of the paediatric surgery department could consist of up to seven parts (Fig. 6, functions 3 to 9), depending on the patient’s age, disease or treatment. The multidisciplinary meeting between health care professionals that took place after the physical follow-up (not in the patient’s presence) was the medium used to exchange professional viewpoints. Interlinkages between professionals whilst a patient was present at the outpatient clinic were few. One interlinkage was observed between the clinical nurse specialist and the paediatric surgeon via brief patient handovers, or when that nurse was physically present while the paediatric surgeon was in consultation with the patient. The order in which professionals saw a patient was not standardised, but was determined by the most efficient schedule. Thus, health care providers involved in the follow-up would see their patients at different stages of the follow-up (they might be the first, second, third or last to see the patient). What previous functions had been performed might be obvious from a patient’s perspective, but complicated to oversee for each individually involved health care provider, even though a schedule for the day was accessible to all involved. In the post-surgery work-as-done, individual care needs of patients and their families were addressed in fragmented ways, with each care provider discussing important medical observations for their field independently. If an aberration in one function occurred, it was dealt with mostly within the discipline in question.

### Building control and feedback loops

Few Control aspects were observed in the surgery work-as-done model. Health care providers were guided by standardised templates in a patient’s electronic health record (EHR). The Follow Me programme of the paediatric surgery department convened a yearly improvement session, attended by all the participating health care providers, patient organisations and the departmental management team, to evaluate outcomes, protocols and organisational indicators.

### Gaps between work-as-imagined and work-as-done models; learning across case studies

In all four work-as-done models, the implemented data collection was aided by templates in the patient EHRs (such as medical assessments) or by standard measurement instruments (such as PROM questionnaires or Bayley Scales of Infant Development), using multiple entry points, EHR modules and software programs. Varying by case studies, the choices regarding data collection on follow-up care mirrored the provision of ‘standard’ care, with the follow-up model of the paediatric surgery department being more specialised and more fragmented, whereas that of the paediatric ICU was more encompassing and less fragmented.

In all four case studies, data were collected and evaluated on medical, developmental and psychosocial outcomes; however, data collection and reporting on the effectiveness of the follow-up was limited. Indicators were missing, for example, to show the number of patients seen for follow-up as a proportion of those that *should* have been seen according to national guidelines or internal protocols. Also missing were indicators providing insights into changes made to treatment plans due to the follow-up programme. Process indicators on the follow-up programme in the studied departments were limited to the number of no-shows (highlighting efficiency in planning and success in informing patients about the importance of the programme); they were not available on the effectiveness of the follow-up programme.

In practice, implementation of a multidisciplinary team approach came in many forms, including (1) a predetermined consecutive order of consultation for the care providers involved (e.g. AMC neonatal ICU and paediatric ICU); (2) access to multiple interdisciplinary data collection points by an individual provider (e.g. paediatric ICU, neonatologist); (3) team-up by providers for data collection (e.g. physiotherapist and neonatologist together in one consultation at the VUMC neonatal ICU); (4) face-to-face patient handovers by paired care providers (neonatologist and psychologist at the paediatric ICU); or (5) a hosting function created to facilitate patient handovers (e.g. VUMC neonatal ICU). Responsibilities for coordinating the follow-up were allocated in different ways in the multidisciplinary teams, for example via the medical assistant (VUMC neonatal ICU), via the lead clinician (AMC neonatal ICU and paediatric ICU) or via the clinical nurse specialist (paediatric surgery).

Yearly evaluation sessions to improve treatment and follow-up, and multiple small control loops to check whether Preconditions were being met, were present in most work-as-done models. However, poor timeliness of control and feedback loops and the lack of process indicators limited the ability of programmes to make adjustments when discrepancies were found. In practice, control and feedback loops operated solely within the scope of the follow-up programmes. This resulted in unplanned interactions with ‘routine care’ outside the department or organisation in almost all work-as-done models (VUMC neonatal ICU, paediatric ICU, paediatric surgery).

## Discussion

This study used the FRAM methodology to evaluate the implementation and organisation of dedicated neonatal and paediatric follow-up care programmes by comparing work-as-done with work-as-imagined. Our findings show that lessons learned from practice show vast data collection for medical, developmental and psychological indicators, a diverse allocation of roles and responsibilities to create a multidisciplinary teams, and many small control and feedback loops. Gaps to be overcome are to address fragmentation of data collection and use; to incorporate and facilitate diversity in multidisciplinary teams in practice; and to include timely actionable indicators to measure effectiveness and performance of the follow-up programme itself.

First, our findings revealed an emphasis on medical, developmental and psychosocial data collection. We believe follow-up programmes can benefit from adding *process indicators* in order to respond in a timely fashion to discrepancies in the follow-up care and to adequately assess effectiveness and performance in the programmes. As it is still largely unknown what follow-up care is most valuable to patient groups in critical paediatric care, it is essential that the effectiveness of follow-up programmes be readily monitored [24]. Previous research has shown the potential of process indicators in situations where outcome indicators lack in actionability [6, 25].

Second, our findings have shown that multidisciplinary teams can take on many forms in practice and that the needs of multidisciplinary teams may differ in different settings. We acknowledge previous research on the importance of incorporating the real-world context (and its people) [26] in order to successfully implement new programmes and advise programme adjustments according to the context and needs of health care professionals and patients.

Our findings also show that the organisation of follow-up care is still based on academic specialisations and superspecialisations which may not align with the broader health, developmental and psychosocial monitoring needed to achieve satisfactory long-term outcomes for critical paediatric patients and their families [27, 28]. Data collection remains fragmented per medical specialisation, diagnosis or treatment group. Our research shows that patients do not remain in these specialisations and require a more holistic approach to manage their care services. Integrated people-centred or person-centred care are ways in which to incorporate this perspective in our care services. Whilst the programme design ensured patient involvement in the development and evaluation of the programme, patient centeredness in the execution still needs attention. The same is seen in the Dutch medical quality registers. Every quality register has been initiated with the respective subinterests in mind, and they contain few interlinkages to other health and social care databases [29]. As treatments and follow-up care by multiple health, social and developmental care providers interrelate with child environmental factors, comprehensive data infrastructures can be important tools for adequately monitoring long-term outcomes.

The measurement of healthcare performance, and the use of healthcare performance data by different end-users (such as providers or patients), are important strategies to improve our health care services and systems in line with the Triple Aim programme for health care change (better health, better patient experience, cost control) [30]. However, responsibilities for long-term patient outcome assessment and follow-up do not appear to be clearly allocated. Further research is needed to understand how follow-up Outcomes can be adjusted to better address the patient perspective and the patients’ broader health, psychosocial and developmental outcomes, instead of mirroring the organisational structures of health care provision.

### Applicability of the FRAM method

The FRAM method required special training and close collaboration between research and parties in practice. This resulted in the strengths and weaknesses listed below. Using direct observations, we were able to view the real-world context by studying the work-as-done, an often overlooked and underreported perspective in research [31].

### Strengths and limitations

A strength of this study was the close collaboration between researchers, the Follow Me project team and the coordinators of the follow-up programmes, resulting in a multidisciplinary, practice-oriented research team. Use of the FRAM methodology with multiple departments implementing similar programmes created cross-case-study learning opportunities. Difficulties in this study included the incorporation of FRAM models into the reflection sessions with the departments, as the methodology was new to most and required some effort to explain. The author VB was trained in the methodology and led the sessions; topics addressed by discussion participants were bountiful and relevant. All study sites were situated in one academic hospital, which may limit generalizability to other hospital settings. Moreover, real practice may still differ from the models developed in this study due to observer bias, and selective sampling of the observations by the departments. To minimize observer bias a close collaboration between researchers, the Follow Me project team and the coordinators of the follow-up programmes, resulting in a multidisciplinary, practice-oriented research team was established. Finally, in order to keep the models readable, our necessary choices regarding foreground and background functions forced us to construct a reduced scope of the ‘real world’. This scope was defined together with the Follow Me project team and resulted in the subsections of the first research question: (a) generating data collection and standardisation for the measurement of long-term outcomes, (b) allocating key stakeholder roles and responsibilities, and (c) building control and feedback loops to aid decision-making. Finally, a limitation of the FRAM method in our study lay in the difference in details observed for the work-as-imagined versus the work-as-done models, although that is an observation on its own. 

## Conclusion

The follow-up programmes of the three critical neonatal and paediatric departments showed vast data collection practices for medical, developmental and psychosocial indicators, a diverse allocation of roles and responsibilities to create multidisciplinary teams, and many small control and feedback loops. Gaps to overcome are to address overspecialisation in data collection, storage and infrastructures; to incorporate and facilitate diversity in multidisciplinary teams in practice; and to include timely actionable indicators to measure effectiveness and performance in the follow-up programme itself. Whilst the programme ensured patient involvement in the development and evaluation of the programme, patient centeredness in the execution still needs attention. The FRAM methodology enabled a structured reflection on the progress of the implementation, based on the observed differences between work-as-imagined and work-as-done in the three teams as well as across the teams.

## Supplementary Information

**Additional file 1.** COREQ checklist

**Additional file 2.** Template observations

**Additional file 3.** Data collected per model

**Additional file 4.** Functions generating data and their aspects

## Data Availability

The digitised data collected and analysed is stored at the Amsterdam UMC and is available upon reasonable request via the corresponding author.

## Abbreviations

AMC: Academic Medical Centre
EHR: Electronic health record
FRAM: Functional resonance analysis method
ICU: Intensive care unit
LNF: Landelijke Neonatale Follow-up
NICU: Neonatal intensive care unit
NVK: Nederlandse Vereniging voor Kindergeneeskunde (Dutch Paediatric Society)
PICU: Paediatric intensive care unit
PROM: Patient-reported outcome measurement
UMC: University Medical Centre
VUMC: VU University Medical Centre Amsterdam
