# Supplementary material for: Implementing structured follow-up of neonatal and paediatric patients: an evaluation of three university hospital case studies using the functional resonance analysis method
Source: BMC Health Serv Res. 2022 Feb 14;22:191. doi: 10.1186/s12913-022-07537-x (PMC8842913; doi:10.1186/s12913-022-07537-x)
Supplement: Supplementary file 1 — Additional file 1. COREQ checklist [file 12913_2022_7537_MOESM1_ESM.pdf]

Implementing structured follow-up of neonatal and paediatric patients: an evaluation of three university hospital case studies using the functional resonance analysis method

Authors

Véronique Bos[1], Daniëlle Roorda[2], Eleonore de Sonnaville[3], Menne van Boven[4], Jaap Oosterlaan[5], Johannes van Goudoever[5], Niek Klazinga[1] and Dionne Kringos[1]

[1] Department of Public and Occupational Health, Amsterdam UMC, University of Amsterdam; and Amsterdam Public Health Research Institute, Amsterdam, Netherlands

[2] Department of Pediatric Surgery, Emma Children's Hospital, Amsterdam UMC, University of Amsterdam and Vrije Universiteit; and Amsterdam Reproduction and Development, Amsterdam, Netherlands

[3] Pediatric Intensive Care Unit, Emma Children's Hospital, Amsterdam UMC, University of Amsterdam, Amsterdam, Netherlands

[4] Neonatal Intensive Care Unit, Emma Children's Hospital, Amsterdam UMC, University of Amsterdam, Amsterdam, Netherlands

[5] Follow Me Programme and Emma Neuroscience Group, Department of Pediatrics, Emma Children's Hospital, Amsterdam UMC, University of Amsterdam; and Amsterdam Reproduction and Development, Amsterdam, Netherlands

Corresponding author

Véronique Bos

Department of Public and Occupational Health

Amsterdam Medical Centre, Amsterdam UMC

University of Amsterdam

Meibergdreef 9

1105 AZ Amsterdam

Netherlands

Email: v.l.bos@amsterdamumc.nl

Additional file 1 – COREQ checklist

Domain 1: Research team and reflexivity

Personal Characteristics

Nr	Item	Guide question/description	
1	Interviewer/facilitator	Which author/s conducted the interview or focus group?	VB
2	Credentials	What were the researcher’s credentials? E.g. PhD, MD	BSc, MA
3	Occupation	What was their occupation at the time of the study?	PhD candidate
4	Gender	Was the researcher male or female?	female
5	Experience and training	What experience or training did the researcher have?	Experienced in interviews for research purposes, FRAM training

Relationship with participants

Nr	Item	Guide question/description	
6	Relationship established	Was a relationship established prior to study commencement?	Before the start of the study, the study and VB were introduced to the teams via an A4 communication and a column in a well-read weekly newsletter of the departments.
7	Participant knowledge of the interviewer	What did the participants know about the researcher? e.g. personal goals, reasons for doing the research	In the communication the background of the interviewer was listed, as well as research goals.
8	Interviewer characteristics	What characteristics were reported about the interviewer/facilitator? e.g. Bias, assumptions, reasons and interests in the research topic	The background of the interviewer in operational management was communicated as well as her affiliation with the hospital environment.

Domain 2: study design

Theoretical framework

Nr	Item	Guide question/description	
9	Methodological orientation and Theory	What methodological orientation was stated to underpin the study? e.g. grounded theory,<br>discourse analysis, ethnography, phenomenology, content analysis	The FRAM was used. The FRAM has a background in the Safety-2 philosophy.

Participant selection

Nr	Item	Guide question/description	

10	Sampling	How were participants selected? e.g. purposive, convenience, consecutive, snowball	Purposive sampling was done, with the request to evaluate the implementation of the Follow Me programme.
11	Method of approach	How were participants approached? e.g. face-to-face, telephone, mail, email	The Follow Me project team contacted the clinical leads of the policlinic per department and observations were scheduled through them.
12	Sample size	How many participants were in the study?	Three departments were included in this study
13	Non-participation	How many people refused to participate or dropped out? Reasons?	All invited interviewees chose to participate in the study. All care providers, patients and their families agreed to the observations for the study when asked beforehand.

Setting

Nr	Item	Guide question/description	
14	Setting of data collection	Where was the data collected? e.g. home, clinic, workplace	The interviews were conducted in the hospital, the observations were conducted in the outpatient clinic. Due to COVID-19 restrictions two evaluation meetings with departments had to be scheduled digitally.
15	Presence of non-participants	Was anyone else present besides the participants and researchers?	no
16	Description of sample	What are the important characteristics of the sample? e.g. demographic data, date	Different age categories of patients were included in the observations reflecting the different content of the programme per age group.

Data collection

Nr	Item	Guide question/description	
17	Interview guide	Were questions, prompts, guides provided by the authors? Was it pilot tested?	The FRAM interview guide from Hollnagel and colleagues was used.
18	Repeat interviews	Were repeat interviews carried out? If yes, how many?	No, however some questions that could not be answered during observations were answered at a later moment in time.

19	Audio/visual recording	Did the research use audio or visual recording to collect the data?	Interviews were audio recorded, observations were not audio/visually recorded.
20	Field notes	Were field notes made during and/or after the interview or focus group?	The field notes of the observations were guided by FRAM template (Additional file 2)
21	Duration	What was the duration of the interviews or focus group?	The duration of the interviews were 15-39 minutes.
22	Data saturation	Was data saturation discussed?	Teams were actively involved in the validation of the findings via improvement sessions in order to capitalize on their daily experience with the programme.
23	Transcripts returned	Were transcripts returned to participants for comment and/or correction?	Transcripts were not made.

Domain 3: analysis and findings

Data analysis

Nr	Item	Guide question/description	
24	Number of data coders	How many data coders coded the data?	One researcher VB coded the data into functions and aspects.
25	Description of the coding tree	Did authors provide a description of the coding tree?	Data use per model is available (Additional file 3), as well as source of data per function and aspect in all models (available upon reasonable request).
26	Derivation of themes	Were themes identified in advance or derived from the data?	All authors agreed on foreground-background coding themes.
27	Software	What software, if applicable, was used to manage the data?	The FRAM Model Visualizer (FMV) software, version 2.1.0, was used to shape the models
28	Participant checking	Did participants provide feedback on the findings?	Yes, improvement sessions were organized with all departments to discuss and provide feedback on the findings.

Reporting

Nr	Item	Guide question/description	
29	Quotations presented	Were participant quotations presented to illustrate the themes / findings? Was each quotation identified? e.g. participant number	No direct quotations were made. All functions and aspects included in the models can be traced back to the source (document,

			interview, or observation) via the research log.
30	Data and findings consistent	Was there consistency between the data presented and the findings?	Yes, data could all be incorporated in the FRAM models which reached consensus with the participants.
31	Clarity of major themes	Were major themes clearly presented in the findings?	Models were used to give a broad overview of work-as-imagined or work-as-done. The themes questioned using the FRAM models were clearly answered by the models.
32	Clarity of minor themes	Is there a description of diverse cases or discussion of minor themes?	Yes, occurrences in the data, however small, were included in the models and discussed in the improvement sessions to validate relevance.
