# Supplementary material for: Implementing structured follow-up of neonatal and paediatric patients: an evaluation of three university hospital case studies using the functional resonance analysis method
Source: BMC Health Serv Res. 2022 Feb 14;22:191. doi: 10.1186/s12913-022-07537-x (PMC8842913; doi:10.1186/s12913-022-07537-x)
Supplement: Supplementary file 2 — Additional file 2. Template observations [file 12913_2022_7537_MOESM2_ESM.pdf]

Implementing structured follow-up of neonatal and paediatric patients: an evaluation of three university hospital case studies using the functional resonance analysis method

Authors

Véronique Bos[1], Daniëlle Roorda[2], Eleonore de Sonnaville[3], Menne van Boven[4], Jaap Oosterlaan[5], Johannes van Goudoever[5], Niek Klazinga[1] and Dionne Kringos[1]

[1] Department of Public and Occupational Health, Amsterdam UMC, University of Amsterdam; and Amsterdam Public Health Research Institute, Amsterdam, Netherlands

[2] Department of Pediatric Surgery, Emma Children's Hospital, Amsterdam UMC, University of Amsterdam and Vrije Universiteit; and Amsterdam Reproduction and Development, Amsterdam, Netherlands

[3] Pediatric Intensive Care Unit, Emma Children's Hospital, Amsterdam UMC, University of Amsterdam, Amsterdam, Netherlands

[4] Neonatal Intensive Care Unit, Emma Children's Hospital, Amsterdam UMC, University of Amsterdam, Amsterdam, Netherlands

[5] Follow Me Programme and Emma Neuroscience Group, Department of Pediatrics, Emma Children's Hospital, Amsterdam UMC, University of Amsterdam; and Amsterdam Reproduction and Development, Amsterdam, Netherlands

Corresponding author

Véronique Bos

Department of Public and Occupational Health

Amsterdam Medical Centre, Amsterdam UMC

University of Amsterdam

Meibergdreef 9

1105 AZ Amsterdam

Netherlands

Email: v.l.bos@amsterdamumc.nl

Additional file 2 - Template observations

<function> :

**Input**	
**Output**	
**Variability of output**	
**Pre-condition**	
**Resources**	
**Control**	
**Time**	
