# Supplementary material for: Implementing structured follow-up of neonatal and paediatric patients: an evaluation of three university hospital case studies using the functional resonance analysis method
Source: BMC Health Serv Res. 2022 Feb 14;22:191. doi: 10.1186/s12913-022-07537-x (PMC8842913; doi:10.1186/s12913-022-07537-x)
Supplement: Supplementary file 3 — Additional file 3. Data collected per model [file 12913_2022_7537_MOESM3_ESM.pdf]

Implementing structured follow-up of neonatal and paediatric patients: an evaluation of three university hospital case studies using the functional resonance analysis method

Authors

Véronique Bos[1], Daniëlle Roorda[2], Eleonore de Sonnaville[3], Menne van Boven[4], Jaap Oosterlaan[5], Johannes van Goudoever[5], Niek Klazinga[1] and Dionne Kringos[1]

[1] Department of Public and Occupational Health, Amsterdam UMC, University of Amsterdam; and Amsterdam Public Health Research Institute, Amsterdam, Netherlands

[2] Department of Pediatric Surgery, Emma Children's Hospital, Amsterdam UMC, University of Amsterdam and Vrije Universiteit; and Amsterdam Reproduction and Development, Amsterdam, Netherlands

[3] Pediatric Intensive Care Unit, Emma Children's Hospital, Amsterdam UMC, University of Amsterdam, Amsterdam, Netherlands

[4] Neonatal Intensive Care Unit, Emma Children's Hospital, Amsterdam UMC, University of Amsterdam, Amsterdam, Netherlands

[5] Follow Me Programme and Emma Neuroscience Group, Department of Pediatrics, Emma Children's Hospital, Amsterdam UMC, University of Amsterdam; and Amsterdam Reproduction and Development, Amsterdam, Netherlands

Corresponding author

Véronique Bos

Department of Public and Occupational Health

Amsterdam Medical Centre, Amsterdam UMC

University of Amsterdam

Meibergdreef 9

1105 AZ Amsterdam

Netherlands

Email: v.l.bos@amsterdamumc.nl

Additional file 3 – Data use per model

Data Work-as-Imagined (code: DOC + #)		
Internal documents (code D + #)	General	1. Follow Me – missie, visie en strategie (Amsterdam UMC)<br>2. Werkwijze Follow Me programma<br>3. Follow Me klinische les polikliniek presentatie<br>4. Follow Me outline<br>5. Presentatie themadag verpleegkundigen Follow Me<br>6. Waarom Follow Me? (Amsterdam UMC)
	NICU	7. Follow Me neonatologie polistafoverleg presentative<br>8. Totaaloverzicht protocollen Follow Me NICU
	PICU	9. Concept follow up protocol IC Kinderen (werkgroep follow me werkgroep IC kinderen)<br>10. Follow Me polikliniek Intensive Care Kinderen; informatie voor ouders (secretariaat van de afdeling Intensive Care Kinderen, locatie Meibergdreef (Emma Kinderziekenhuis))
	Paediatric surgery	11. Follow Me Polikliniek Aangeboren Aandoeningen; informatie voor ouders (Follow Me casemanagers)
External documents (code D + #)	General	12. Follow Me programma poster
	NICU	13. Aanbevelingen Landelijke Neonatale Follow-up – NICU follow-up (2015, werkgroep Landelijke Neonatale Follow-up)<br>14. www.landelijkeneonatalefollowup.nl
	PICU	15. Richtlijn follow-up van kinderen na opname op een intensive care (2017, SICK, NVK)
	Paediatric surgery	16. Sturen op Kwaliteit Kinderchirurgie - Ontwikkeling van een kernset kwaliteitsindicatoren voor de behandeling en follow-up van aangeboren aandoeningen binnen de kinderchirurgie (2018, NFU)
Interviews Work-as-Imagined (code INT + #)	General	Steering group Follow Me:<br>1. Director van het Follow Me programme & Steering group member Follow Me programme<br>2. Project coordinator Follow Me programme & Steering group member Follow Me programme<br>3. Steering group member Follow Me programme<br>4. Steering group member Follow Me programme
	NICU	5. Coordinating PhD Follow Me NICU<br>6. Head of department NICU
	PICU	7. Coordinating PhD Follow Me PICU<br>8. Head of department PICU
	Paediatric surgery	9. Coordinating PhD Follow Me paediatric surgery<br>10. Head of department paediatric surgery

Data Work-as-Done		
Observations (code OBS + #)	NICU location Meibergdreef	1. 28th October 2019 – neonatologist & paediatric psychologist<br>2. 21st November 2019 – neonatologist
	NICU location Boelelaan	3. 1st November 2019 –psychologist, neonatologist, physiotherapist

		4. 19th November 2019 – doctor's assistant, neonatologist, physiotherapist
	PICU	5. 27th September 2019 –paediatric intensivist, psychologist, revalidation specialist, paediatric pulmonologist.<br>6. 11th October 2019 – paediatric intensivist
	Paediatric surgery	7. 8th October 2019 –paediatric surgeon, PhD coordinator Follow Me programme PSU, physiotherapists specialized in children development, specialized nurse)<br>8. 25th October 2019 – paediatric surgeon, physiotherapist specialized in paediatric development)
Department meetings (code M + #)	NICU	1. 25th May 2020 – FRAM evaluation session for both locations together
	PICU	2. 28th May 2020 – yearly evaluating outcomes follow me PICU and FRAM evaluation<br>3. 6th July 2020 – reflecting on evaluating session and decide on areas for improvement
	Paediatric surgery	4. 27th January 2020 – yearly evaluating outcomes follow me paediatric surgery and FRAM evaluation<br>5. 9th March 2020 – reflecting on evaluating session and decide on areas for improvement
Documents (code D + #)	NICU	-
	PICU	17. Presentation 'Verbetersessie 2020 intensive care kinderen'
	Paediatric surgery	18. Outcomes patient satisfaction survey Follow Me paediatric surgery
