# Supplementary material for: Implementing structured follow-up of neonatal and paediatric patients: an evaluation of three university hospital case studies using the functional resonance analysis method
Source: BMC Health Serv Res. 2022 Feb 14;22:191. doi: 10.1186/s12913-022-07537-x (PMC8842913; doi:10.1186/s12913-022-07537-x)
Supplement: Supplementary file 4 — Additional file 4. Functions generating data and their aspects [file 12913_2022_7537_MOESM4_ESM.pdf]

Implementing structured follow-up of neonatal and paediatric patients: an evaluation of three university hospital case studies using the functional resonance analysis method

Authors

Véronique Bos[1], Daniëlle Roorda[2], Eleonore de Sonnaville[3], Menne van Boven[4], Jaap Oosterlaan[5], Johannes van Goudoever[5], Niek Klazinga[1] and Dionne Kringos[1]

[1] Department of Public and Occupational Health, Amsterdam UMC, University of Amsterdam; and Amsterdam Public Health Research Institute, Amsterdam, Netherlands

[2] Department of Pediatric Surgery, Emma Children's Hospital, Amsterdam UMC, University of Amsterdam and Vrije Universiteit; and Amsterdam Reproduction and Development, Amsterdam, Netherlands

[3] Pediatric Intensive Care Unit, Emma Children's Hospital, Amsterdam UMC, University of Amsterdam, Amsterdam, Netherlands

[4] Neonatal Intensive Care Unit, Emma Children's Hospital, Amsterdam UMC, University of Amsterdam, Amsterdam, Netherlands

[5] Follow Me Programme and Emma Neuroscience Group, Department of Pediatrics, Emma Children's Hospital, Amsterdam UMC, University of Amsterdam; and Amsterdam Reproduction and Development, Amsterdam, Netherlands

Corresponding author

Véronique Bos

Department of Public and Occupational Health

Amsterdam Medical Centre, Amsterdam UMC

University of Amsterdam

Meibergdreef 9

1105 AZ Amsterdam

Netherlands

Email: v.l.bos@amsterdamumc.nl

Additional file 4 – Functions generating data and their aspects

Table 1: Work-as-Imagined Follow Me programme - functions generating follow-up data

Function	Aspects (**I**nput, **O**utput, **P**recondition, **R**esource, **C**ontrol, and **T**ime)
2) to collect PROMs	I: Invite to questionnaire(s)<br>**O: PROMs collected**<br>P: Questionnaires accessible in KLIK software or 'Mijn Dossier' (patient portal in electronic health record), patient access to KLIK or 'Mijn Dossier' (all preconditions modelled as accessible questionnaires)<br>R: KLIK, 'Mijn Dossier' (modelled as EHR), patient (from age 8 years) or parents (parent proxy measurements)<br>C: none observed<br>T: 2 weeks before appointment at outpatient standardized follow-up programme
3) to conduct outpatient follow-up	I: schedule<br>**O: standardized multidisciplinary follow-up patient data collected**, new initiatives, referrals further individual treatment, knowledge about long term outcomes of individual patients treated<br>P: financing, commitment involved care professionals, support hospital, support IT, workspace<br>R: EHR (EPIC questionnaires, report of the day, 'Smart text'),<br>C: completeness of data, multidisciplinary team (present in multidisciplinary meeting), protocol per department and condition/disease/treatment infant/child, potential complaints, ad hoc interventions needed by project team, expertise centrums, follow-up %, patient satisfaction, word of mouth patient families<br>T: last follow-up 17 years of age, standardized times to be comparable (both modelled as protocol)
4) to adjust treatment or guidance	I: referral from follow Me<br>**O: Quality of life outcomes after adjusted treatment due to follow-up**<br>P: none observed<br>R: none observed<br>C: none observed<br>T: preventive/ timely (asap) proactive due to follow up

Table 2: Work-as-Done functions of the neonatal ICU, location Meibergdreef, generating follow-up data

Function	Aspects (**I**nput, **O**utput, **P**recondition, **R**esource, **C**ontrol, and **T**ime)
2) to collect PROMs	I: invite<br>**O: PROMs**<br>P: none observed<br>R: none observed<br>C: reminder about two weeks in advance<br>T: none observed
3) to conduct follow-up programme (clinician)	I: patient at outpatient clinic<br>O: consent form for research purposes<br>O: informed patient/family about motor development, growth development, general wellbeing and potential medical complaints<br>**O: patient data registered on motor development, growth development, general wellbeing and medical complaints**<br>O: handover to psychologist<br>O: referral to other medical treatment when necessary<br>P: schedule<br>R: pre-scripted questions in EHR on general wellbeing, movement assessment battery for children (OBS1), Movement ABC (OBS2), scale and measuring tape, EHR, trained medical doctors specialized in neonatal check ups<br>C: none observed<br>T: takes about 1.5 hours (patient (child) asks parents how much longer it will take)

4) to conduct follow-up programme (psychologist)	I: patient at outpatient clinic<br>**O: patient data on cognitive development,** referral when necessary<br>P: schedule<br>R: trained psychologist, PROMs, informed psychologist on clinical follow up, BSID-III-NL (24 months of age), WPPSI-II-NL (5,5 years of age), WISC-V-NL (8 years of age)<br>C: none observed<br>T: duration 1.5-2 hours

Table 3 Work-as-Done functions neonatal ICU, location Boelelaan, generating follow-up data

Function	Aspects (**I**nput, **O**utput, **P**recondition, **R**esource, **C**ontrol, and **T**ime)
3) to collect PROMs	I: invite<br>O: **paper based PROMs**<br>P: none observed<br>R: paper based questionnaires ('Lexilijst Nederlands', behavioural questionnaire for children 1.5 until 5 years of age)<br>C: none observed<br>T: none observed
6) to conduct follow-up (psychologist)	I: patient in waiting room (digital cue in EHR scheduling module, modelled as EHR)<br>O: **patient data on cognitive development on paper**, refer to desk to check in for further follow up<br>P: schedule<br>R: CBCL/'lexilijsten', patient summary, paper checklist, EHR, trained medical psychologist in neonatal follow-up, Bayley scales of infant development<br>C: none observed<br>T: none observed
9) to weigh and measure patient (doctor's assistant)	I: patient at outpatient clinic<br>O: handover from doctor's assistant, **patient data on growth development in EHR**<br>P: none observed<br>R: scale, measuring tape, EHR, trained doctor's assistant, patient, general questionnaire in EHR (modelled as EHR)<br>C: none observed<br>T: none observed
10) to conduct follow-up (neonatologist)	I: patient in waiting room (digital cue in EHR scheduling module) and handover from doctor's assistant<br>O: **patient data registered on growth development (when doctor's assistant is absent), general wellbeing and medical complaints**<br>P: schedule<br>R: EHR, trained specialized in neonatal care, scale and measuring tape, patient summary<br>C: none observed<br>T: none observed
11) to conduct follow-up (physiotherapist)	I: coordination doctor's assistant<br>O: **patient data registered on motor development on paper**, feedback patient on motor development<br>P: schedule<br>R: trained physiotherapist specialized in neonatal follow-up, patient summary, TOP results (physiotherapy primary care), Alberta infant motor scale<br>C: none observed<br>T: none observed

Table 4 Work-as-Done functions paediatric ICU generating follow-up data

Function	Aspects (**I**nput, **O**utput, **P**recondition, **R**esource, **C**ontrol, and **T**ime)
2) to collect PROMs	I: invite (to KLIK system)<br>O: **PROMs in KLIK system**<br>P: none observed<br>R: KLIK system containing questionnaires<br>C: none observed<br>T: none observed

3) to weigh and measure patient (doctor's assistant)	I: patient at outpatient clinic<br>O: **patient data on weight, blood pressure and measurement in electronic health record (EHR)**<br>P: none observed<br>R: scale, measuring tape, EHR, trained doctors assistant, patient, blood pressure measurement instrument<br>C: call from doctor in the case of no show, to offer standardized follow-up programme (clinician) check on measurements<br>T: none observed
5) to conduct follow-up (paediatric intensivist)	I: patient in waiting room<br>O: **patient data registered on general wellbeing, medical complaints, cognitive and motor development**<br>O: check on weight and measure patient<br>O: referral<br>P: schedule<br>R: EHR<br>C: none observed<br>T: approx. 30 min per consult per patient (2.5 hours for neuropsychological consult)
7) to conduct follow-up (psychologist)	I: hand-over from clinician<br>O: **referral, EHR free text**<br>P: none observed<br>R: PROMs, trained medical psychologist<br>C: none observed<br>T: none observed

Table 5 Work-as-Done functions paediatric surgery generating follow-up data

Function	Aspects
2) to collect PROMs	I: invite to KLIK system, invite to 'Mijn dossier' questionnaire<br>O: **PROMs on psychosocial indicators in KLIK system, PROMs on medical indicators in EHR**<br>P: none observed<br>R: KLIK programme, EHR patient portal access<br>C: none observed<br>T: none observed
3) to weigh and measure patient (doctor's assistant)	I: patient at outpatient clinic<br>O: **patient data on weight and measurement in EHR**<br>P: schedule<br>R: scale, measuring tape, EHR, trained doctor's assistant, patient<br>C: none observed<br>T: none observed
4) to conduct follow-up (specialized nurse)	I: schedule<br>O: **patient data on general wellbeing**<br>P: patient present in waiting room<br>R: patient data on weight and measurement in EHR, interpreter phone, template general wellbeing questions, trained nurse<br>C: none observed<br>T: none observed
5) to conduct follow-up (paediatric surgeon)	I: schedule<br>O: **patient data on medical indicators in EHR**<br>P: patient present in waiting room<br>R: patient, HER<br>C: none observed<br>T: none observed
6) to conduct follow-up (paediatric gastroenterologist; when indicated)	I: schedule<br>O: **patient data on gastrointestinal development in EHR** (modelled as patient data on medical indicators)<br>P: patient present in waiting room<br>R: interpreter phone, EHR, trained paediatric gastroenterologist<br>C: none observed<br>T: none observed

7) to conduct follow-up (paediatric pulmonologist; when indicated)	I: schedule<br>O: **patient data on pulmonological development in EHR** (modelled as patient data on medical indicators)<br>P: patient present in waiting room<br>R: interpreter phone, EHR, trained paediatric pulmonologist<br>C: none observed<br>T: none observed
8) to conduct follow-up (physiotherapist)	I: schedule<br>O: **patient data on motor development in EHR**<br>P: patient in waiting room<br>R: Alberta infant motor scales (AIMS), Bayley Scale of Infant Development (BSID), EHR, physiotherapist<br>C: none observed<br>T: none observed
9) to conduct follow-up (medical psychologist)	I: schedule<br>O: none observed<br>P: patient in waiting room<br>R: PROMs, KLIK system, EHR<br>C: none observed<br>T: none observed
